# Functional Characterization of an Interferon Gamma Receptor-Like Protein on Entamoeba histolytica

**DOI:** 10.1128/IAI.00540-19

**Published:** 2019-10-18

**Authors:** Julieta Pulido-Ortega, Patricia Talamás-Rohana, Martín Humberto Muñoz-Ortega, Liseth Rubí Aldaba-Muruato, Sandra Luz Martínez-Hernández, María del Rosario Campos-Esparza, Daniel Cervantes-García, Aralia Leon-Coria, France Moreau, Kris Chadee, Javier Ventura-Juárez

**Affiliations:** a Departamento de Morfología, Centro de Ciencias Básicas, Universidad Autónoma de Aguascalientes, Aguascalientes, Ags., Mexico; b Departamento de Infectómica y Patogénesis Molecular, Centro de Investigación y de Estudios Avanzados del Instituto Politécnico Nacional, Mexico City, Mexico; c Departamento de Química, Centro de Ciencias Básicas, Universidad Autónoma de Aguascalientes, Aguascalientes, Ags., Mexico; d Unidad Académica Multidisciplinaria Zona Huasteca, Universidad Autónoma de San Luis Potosí, San Luis Potosí, S.L.P., Mexico; e CONACYT-Departamento de Microbiología, Centro de Ciencias Básicas, Universidad Autónoma de Aguascalientes, Aguascalientes, Ags., Mexico; f Department of Microbiology, Immunology and Infectious Diseases, Snyder Institute for Chronic Diseases, Cumming School of Medicine, University of Calgary, Calgary, Alberta, Canada; University of Pennsylvania

**Keywords:** *Entamoeba histolytica*, amoebiasis, cysteine proteases, IFN-γ, erythrophagocytosis, cytopathic effect

## Abstract

Entamoeba histolytica is an anaerobic parasitic protozoan and the causative agent of amoebiasis. E. histolytica expresses proteins that are structurally homologous to human proteins and uses them as virulence factors. We have previously shown that E. histolytica binds exogenous interferon gamma (IFN-γ) on its surface, and in this study, we explored whether exogenous IFN-γ could modulate parasite virulence. We identified an IFN-γ receptor-like protein on the surface of E. histolytica trophozoites by using anti-IFN-γ receptor 1 (IFN-γR1) antibody and performing immunofluorescence, Western blot, protein sequencing, and *in silico* analyses. Coupling of human IFN-γ to the IFN-γ receptor-like protein on live E. histolytica trophozoites significantly upregulated the expression of E. histolytica cysteine protease A1 (*Eh*CP-A1), *Eh*CP-A2, *Eh*CP-A4, *Eh*CP-A5, amebapore A (APA), cyclooxygenase 1 (*Cox-1*), Gal-lectin (*Hgl*), and peroxiredoxin (*Prx*) in a time-dependent fashion. IFN-γ signaling via the IFN-γ receptor-like protein enhanced E. histolytica’s erythrophagocytosis of human red blood cells, which was abrogated by the STAT1 inhibitor fludarabine. Exogenous IFN-γ enhanced chemotaxis of E. histolytica, its killing of Caco-2 colonic and Hep G2 liver cells, and amebic liver abscess formation in hamsters. These results demonstrate that E. histolytica expresses a surface IFN-γ receptor-like protein that is functional and may play a role in disease pathogenesis and/or immune evasion.

## Abstract (precis)

Entamoeba histolytica is an anaerobic parasitic protozoan and the causative agent of amoebiasis. E. histolytica expresses proteins that are structurally homologous to human proteins and uses them as virulence factors. We have previously shown that E. histolytica binds exogenous interferon gamma (IFN-γ) on its surface, and in this study, we explored whether exogenous IFN-γ could modulate parasite virulence.

## INTRODUCTION

Entamoeba histolytica infects the human large intestine, causing amoebiasis, dysentery, and in advanced cases, amoebic liver abscesses, leading to ∼55,000 deaths annually (1). Studies on the host-parasite relationship have established that E. histolytica has developed strategies to escape from host immune responses (2–5). This phenomenon is known as positive natural selection, which drives the increase in prevalence of advantageous traits, and it has played a central role in the development of E. histolytica as a human parasite (6). On the other hand, it could be taken as coevolution at the biochemical level, defined as the process of reciprocal, adaptive genetic change between interacting species (7). In the colon, E. histolytica trophozoites overcome innate host defenses with molecules such as cysteine proteases to degrade mucus (8) and prostaglandin E_2_ (PGE_2_) to stimulate epithelial cells to produce interleukin-8 (IL-8), a chemoattractant for neutrophils (9). Various virulence factors act on different stages of the invasion process, such as Gal/GalNAc lectin for adhesion to host cells (10), amebapores that cause cytolysis of immune cells (11), and cysteine proteases that degrade mucins (12) and immunoglobulins like IgA (13) and cause tissue destruction (14).

The ability of E. histolytica trophozoites to survive in the host has been related in part to the remarkable mobility of surface antigens after interaction with antibodies, which leads to elimination of the resulting antigen-antibody complexes by capping (15). E. histolytica trophozoites can protect themselves from reactive oxygen species (ROS) produced by neutrophils with peroxiredoxin, a 29-kDa surface protein that has potent antioxidant activity (16, 17). In addition, E. histolytica trophozoites express a protein similar to CD59 on the cell membrane that prevents the polymerization of complement protein C9 (18). E. histolytica also expresses a 55-kDa protein, similar to the extracellular loop of human occludin, which allows it to bind to intestinal epithelial cells (19). Furthermore, through bioinformatics analysis, two proteins, a GTPase of the Rab family and a thioredoxin containing a TIR-like domain similar to those of the IL-1 receptor and human Toll-like receptors (TLRs), have been identified (20). Reports have also shown that E. histolytica trophozoites bind the inflammatory cytokine IL-8 through a 29-kDa membrane-associated protein, triggering chemotaxis of the parasites to the source of IL-8 (21). *In vitro* chemotaxis assays, utilizing gradients of tumor necrosis factor alpha (TNF-α), have shown that E. histolytica is attracted toward the source of this cytokine (22).

Previous studies have shown that interferon gamma (IFN-γ) binds on the surface of E. histolytica and reduces protein and DNA synthesis in cultured E. histolytica trophozoites (23). We have shown that E. histolytica trophozoites derived from colonic tissues from fulminant amoebic colitis patients are highly positive for IFN-γ (24) and speculated that E. histolytica has a surface binding protein for this cytokine. In this study, we demonstrate that IFN-γ coupling to E. histolytica IFN-γ receptor-like protein upregulated virulence factors that enhanced phagocytosis, cytopathic effects on colonic and liver cells, and liver abscess formation in a hamster model.

## RESULTS

### Detection of IFN-γ on E. histolytica.

**FIG 1:**
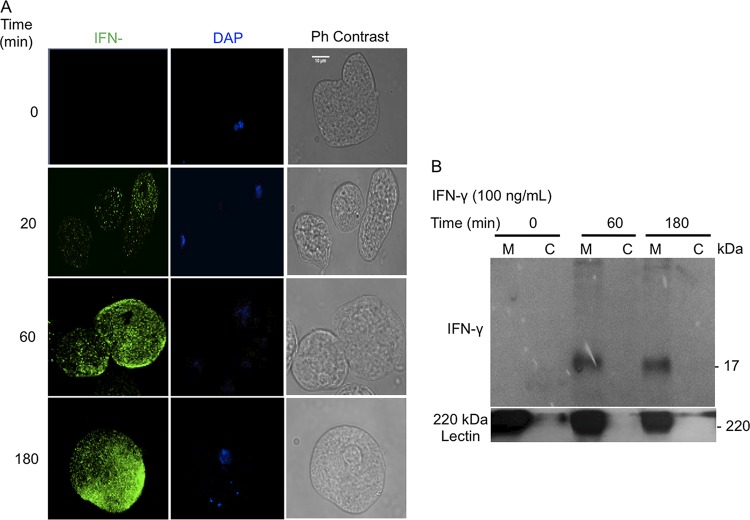
Localization of IFN-γ on the surface of E. histolytica trophozoites. (A) Immunodetection of the IFN-γ protein on the surface of E. histolytica trophozoites incubated with anti-human IFN-γ antibody (1:100 dilution), followed by a secondary antibody conjugated to Alexa 488 (1:1,000), imaged by confocal microscopy. Note that the intense green staining is absent in the control (without IFN-γ) at time zero. Ph contrast, phase-contrast images. (B) Western blot detection of the 17-kDa protein corresponding to IFN-γ on E. histolytica membrane (M) and cytoplasmic (C) fractions at 60 and 180 min. The 220-kDa lectin was used as an internal control to confirm membrane localization and subcellular fractionation. Scale bar represents 10 μm.

IFN-γ plays an important role in host defense against E. histolytica by activating macrophages to produce ROS and nitrogen species that are cytotoxic to the parasite (16). With the use of a highly specific anti-human IFN-γ monoclonal antibody, we detected by immunofluorescence increasing amounts of IFN-γ–antibody complexes on the surface of E. histolytica trophozoites at 60 and 180 min of exposure (Fig. 1A). To support the specific recognition of IFN-γ, a Western blot analysis was performed under reducing conditions that identified a 17-kDa protein corresponding to IFN-γ localized on the membrane of E. histolytica trophozoites (Fig. 1B). As a positive control, an antibody against the Gal/GalNAc lectin (4) was used to confirm the localization of IFN-γ on the E. histolytica membrane (Fig. 1B).

**FIG 2:**
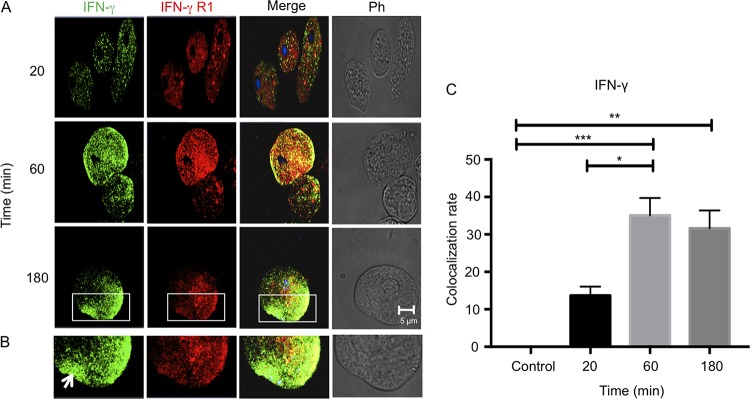
Colocalization of IFN-γ and IFN-γ receptor-like protein on E. histolytica trophozoites. E. histolytica trophozoites were incubated with IFN-γ for 20, 60, or 180 min and analyzed by confocal microscopy. After the interaction, trophozoites were incubated with a polyclonal anti-IFN-γ antibody (1:100 dilution) and a monoclonal anti-IFN-γR1 antibody (1:100 dilution), followed by Alexa Fluor 594- and Alexa Fluor 488-labeled secondary antibodies, respectively. (A) Micrographs show that IFN-γ bound to the surface of E. histolytica trophozoites; an increase in positive signal was obtained with longer exposure time. The label for IFN-γ receptor-like protein was constant after 3 h of interaction and was displaced to the uroid side of trophozoites. Increased colocalization at 1 h of interaction was observed. (B) Images illustrating morphology that demonstrate capping (arrow) on an E. histolytica trophozoite after interaction with IFN-γ for 3 h. Ph, phase-contrast images. (C) Analysis was performed according to the Costes methodology, which includes the elimination of autofluorescence with the Coloc2 plugin of Fiji software (ImageJ). The graph was made with GraphPad Prism 7. *, *P* < 0.05; **, *P* < 0.01; ***, *P* < 0.001.

**FIG 3:**
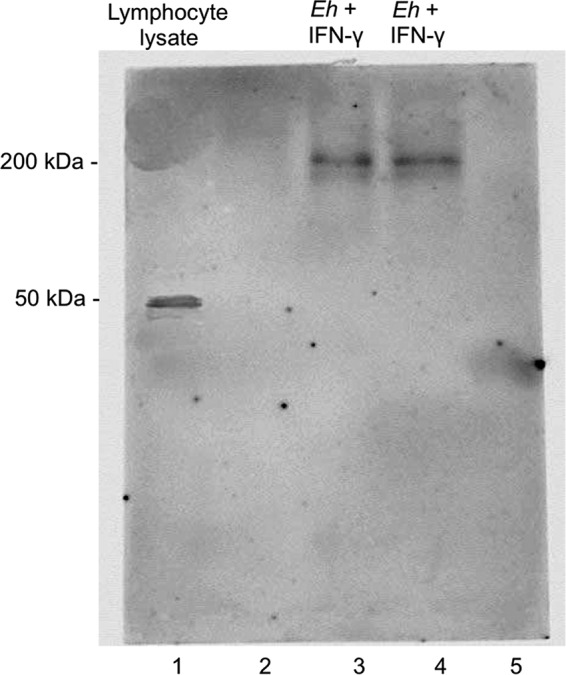
Western blot of E. histolytica membrane fractions using anti-human IFN-γR1 antibody. E. histolytica proteins were analyzed by 12% SDS-PAGE and immunoblotting. E. histolytica trophozoites were incubated with or without 100 ng/ml IFN-γ. Note strong immunoreactivity with the anti-IFN-γR1 antibody at 200 kDa in E. histolytica membrane fractions (lanes 3 and 4) and at 50 kDa in lymphocyte lysate used as controls (lane 1). Lanes 2 and 5 are blanks.

To quantify the localization of IFN-γ to the putative receptor on the surface of E. histolytica trophozoites, parasites were incubated with anti-human IFN-γR1 monoclonal antibody and anti-IFN-γ polyclonal antibody and binding quantified by immunofluorescence. Surprisingly, we observed for both antibodies (anti-IFN-γR1 and anti-IFN-γ antibodies) strong signals as early as 20 min that increased in intensity after 180 min (Fig. 2A) toward the uroid end of the E. histolytica trophozoites (Fig. 2B, white arrow). Colocalization of IFN-γ and anti-human IFN-γR1 antibody on trophozoites was highest after 60 min of interaction with IFN-γ (Fig. 2C). To corroborate the results obtained by confocal microscopy, membrane proteins from trophozoites were separated by SDS-PAGE (12%) and subjected to Western blotting with a monoclonal antibody against IFN-γR1 (trophozoites were incubated for 20 min with recombinant human IFN-γ). As a positive control, the antibody recognized IFN-γR1 in human leukocyte lysates at 50 kDa (Fig. 3, lane 1). Our results suggest that E. histolytica trophozoites express a membrane protein of approximately 200 kDa that is recognized by anti-human IFN-γR1 antibody independently of whether the trophozoites are exposed to exogenous IFN-γ or not (Fig. 3). These results suggest that E. histolytica expresses an IFN-γ receptor-like molecule on its surface that binds exogenous IFN-γ on the surface of the parasite.

### Mass spectrometry analysis.

**FIG 4:**
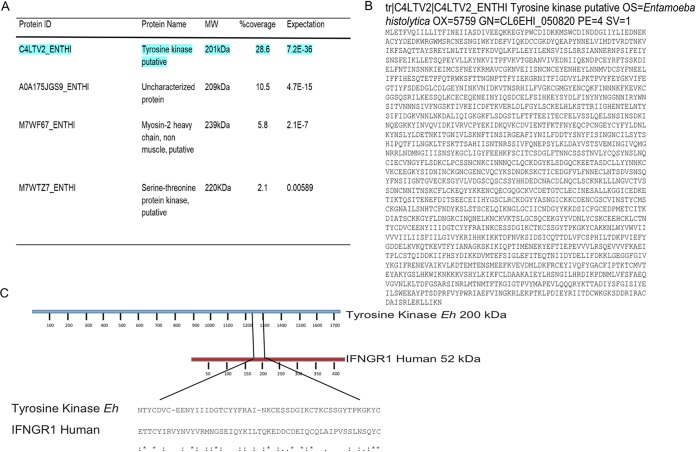
Analysis of the anti-human IFN-γR1 antibody detected a 200-kDa amebic protein. (A) Analysis of the 200-kDa-protein band by mass spectrometry (MS) identified four E. histolytica proteins by peptide analysis. The highlighted protein at the top of the list is reported as a putative amebic surface antigen and as a putative tyrosine kinase of E. histolytica. (B) Sequence of the reported E. histolytica 200-kDa surface antigen (putative tyrosine kinase). (C) Alignment of IFN-γR1 human amino acid sequence and E. histolytica 200-kDa surface antigen (putative tyrosine kinase). The similar amino acid motifs, with 20% identity between the two proteins, are flanked by slanted lines. Ten of the amino acids in this motif are identical, and the other 20 have similar physicochemical properties.

To identify the 200-kDa protein, extracts of E. histolytica membranes were separated by SDS-PAGE and stained with Coomassie blue. The 200-kDa protein was then excised and analyzed by mass spectrometry (MS). Peptide analysis revealed four proteins in the E. histolytica proteome that matched with different scores (Fig. 4A). The putative surface antigen C4LTV2_ENTHI, reported as a putative tyrosine kinase, showed the highest match and percent coverage in comparison with the protein with the expected molecular weight of approximately 200 kDa (Fig. 4B). These peptides cover 28.6% of the amebic 200-kDa-protein sequence reported as a putative tyrosine kinase. Due to the high score and other features of the C4LTV2_ENTHI sequence, our results indicate a high possibility that the anti-IFN-γR1 antibody detected the amebic protein in the Western blot and is most likely the putative tyrosine kinase. Further investigation of this possibility showed (Fig. 4C) that the human IFN-γR1 amino acid sequence has an ETTCYIRVYNVYVRMNGSEIQYKILTQKEDDCDEIQCQLAIPVSSLNSQYC motif in the third extracellular loop, characterized as a binding site for IL-8, which aligns with the NTYCDVCEENYIIIDGTCYYFRAINKCESSDGIKCTKCSSGYTPKGKYC motif of the 200-kDa amebic surface antigen (putative tyrosine kinase), sharing identical amino acids at 10 specific positions, while the other 20 amino acids, although not identical, have similar physicochemical properties.

### IFN-γ upregulates the expression of E. histolytica virulence factors.

**FIG 5:**
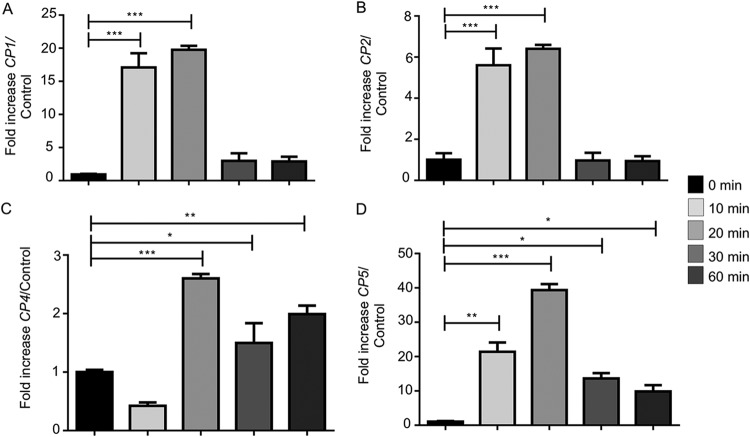
IFN-γ upregulated cysteine protease expression in E. histolytica trophozoites. (A to D) E. histolytica trophozoites treated or not with IFN-γ for 10, 20, 30, or 60 min showed increased expression of cysteine protease genes associated with E. histolytica pathogenicity. Note maximal upregulation of *EhCP-A1*, *EhCP-A2*, *EhCP-A4*, and *EhCP-A5* expression after 20 min of IFN-γ stimulation. *, *P* < 0.05; **, *P* < 0.01; ***, *P* < 0.001.

**FIG 6:**
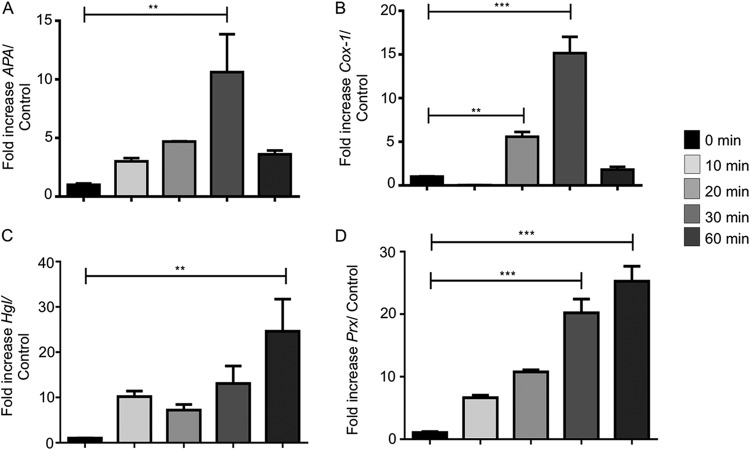
IFN-γ upregulated other virulence genes in E. histolytica in a temporal fashion. (A to D) Interaction of E. histolytica trophozoites with IFN-γ upregulated *APA*, *Cox-1*, *Hgl*, and *Prx* (peroxiredoxin), with maximal expression occurring after 30 min. *, *P* < 0.05; **, *P* < 0.01; ***, *P* < 0.001.

Reverse transcriptase quantitative PCR was performed to determine if exposure of live E. histolytica trophozoites to IFN-γ could modulate the transcription of key virulence factors in E. histolytica cells. After 20 min of exposure to IFN-γ, E. histolytica cysteine protease A1 (*EhCP-A1*), *EhCP-A2*, *EhCP-A4*, and *EhCP-A5* mRNAs were significantly increased compared to their levels in control E. histolytica trophozoites in the absence of IFN-γ. The fold increases were highest for *EhCP-A1* and *EhCP-A5* (20- and 39-fold, respectively) (Fig. 5A and D). E. histolytica amebapore A (*APA*), cyclooxygenase 1 (*Cox-1*), and peroxiredoxin (*Prx*) mRNAs were significantly increased, by 16-fold, after 30 min of exposure (Fig. 6A and B). The mRNA expression of both *Prx* (20-fold) and Gal-lectin (*Hgl*) (17-fold) peaked after 60 min (Fig. 6C and D).

### Pretreating E. histolytica trophozoites with IFN-γ increases erythrophagocytosis.

**FIG 7:**
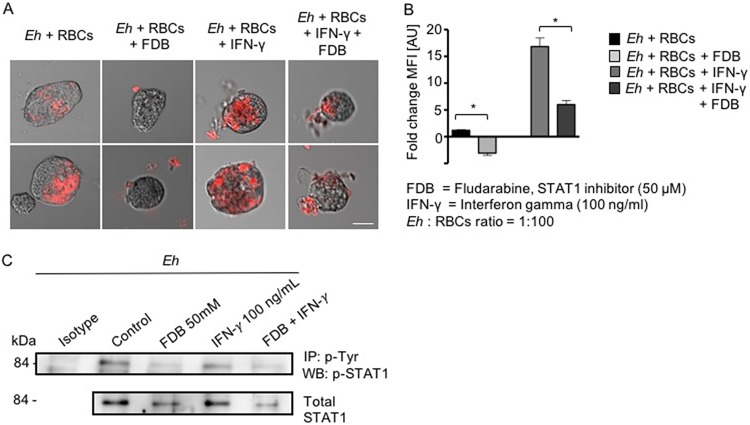
IFN-γ enhanced erythrophagocytosis in E. histolytica trophozoites. (A) Images of erythrophagocytosis by nontreated E. histolytica trophozoites (*Eh*) or by trophozoites in the presence of fludarabine or IFN-γ or both. Note that IFN-γ-stimulated erythrophagocytosis was substantially inhibited with the STAT1 inhibitor fludarabine. Red fluorescence shows ingested erythrocytes. Scale bar represents 10 μm. (B) Histogram showing quantification of the fluorescence of erythrocytes phagocytosed in the different assays. MFI, mean fluorescence intensity; AU, arbitrary units. *, *P* < 0.05. (C) Basal and IFN-γ-induced phosphorylation of STAT1 in E. histolytica is inhibited by the phospho-STAT1 inhibitor fludarabine. IP, immunoprecipitation; WB, Western blotting.

One of the properties of E. histolytica is its extraordinary phagocytic activity against a variety of particulate materials, including bacteria, sloughed epithelial cells, and erythrocytes. The latter activity has been widely accepted as a characteristic presented by invasive E. histolytica (25). For this reason, we determined whether IFN-γ enhanced erythrophagocytosis in E. histolytica trophozoites. IFN-γ pretreatment for 20 min significantly increased trophozoite phagocytosis of red blood cells (Fig. 7A), which was specifically abrogated by the phospho-STAT1 inhibitor fludarabine (Fig. 7B). These results indicate that IFN-γ promoted erythrophagocytosis when it bound to E. histolytica trophozoites through the IFN-γ receptor-like protein, possibly by activating STAT1. Accordingly, we next investigated whether IFN-γ-stimulated E. histolytica phosphorylated STAT1. To investigate this, we immunoprecipitated E. histolytica proteins with phospho-tyrosine and performed Western blotting with phospho-STAT1 under the conditions shown in Fig. 7B. Surprisingly, the basal expression (nonstimulated controls) of phospho-STAT1 in E. histolytica trophozoites was similar to that in IFN-γ-stimulated trophozoites, and pretreatment with the phospho-STAT1 inhibitor fludarabine (50 μM) markedly decreased phospho-STAT1 (Fig. 7C). The viability of E. histolytica trophozoites treated with 50 mM fludarabine was >95% as determined by the trypan blue exclusion assay. Lower concentrations of fludarabine (10 and 25 nM) were not effective in inhibiting basal IFN-γ-stimulated phospho-STAT1. These results demonstrate that basal expression of STAT1 in E. histolytica trophozoites was constitutively high and that STAT1 could not be phosphorylated further with IFN-γ. However, under both conditions, fludarabine markedly inhibited the phosphorylation of STAT1 (Fig. 7C).

### Pretreating E. histolytica trophozoites with IFN-γ increases chemotaxis.

**FIG 8:**
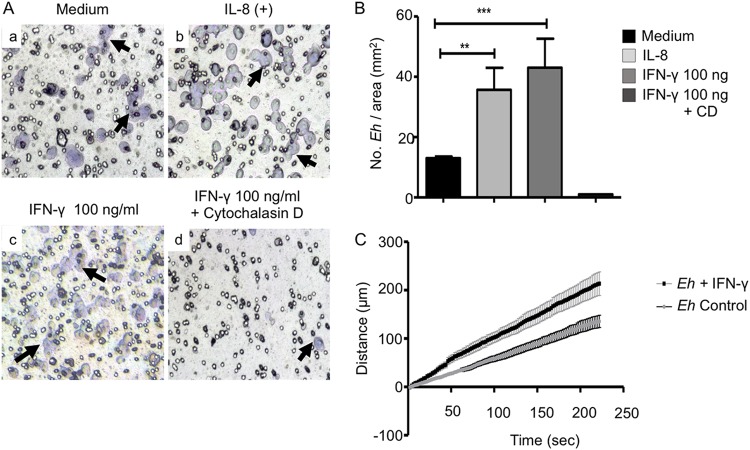
Chemotaxis of E. histolytica trophozoites toward human IFN-γ. (A) Microscopy images of E. histolytica trophozoites that were deposited in the upper chambers of Transwell units and allowed to migrate to the lower chambers in the absence (a) or presence (c) of IFN-γ or in the presence of IL-8 as a positive migration control (b) or of IFN-γ in the presence of 10 μg/ml of cytochalasin D (d). Arrows indicate E. histolytica trophozoites. (B) Numbers of E. histolytica trophozoites/mm^2^. Data represent values from four independent experiments. CD, cytochalasin D. (C) E. histolytica trophozoite velocities tracked in the presence or absence of IFN-γ. The graph represents migration velocities recorded by real-time video microscopy. **, *P* < 0.01; ***, *P* < 0.001.

Chemotaxis is a phenomenon in which cells direct their movements according to the presence of certain chemical substances in the environment. E. histolytica orients its migration or chemotaxis based on external stimuli like serum proteins, bacteria, or molecules released by epithelial and immune cells (26), such as IL-8 (21). This activity in E. histolytica has been correlated with its invasive capacity (21, 27). Chemotaxis requires specific recognition between the chemoattractant and the receptor on the cell, leading to a series of activation signals that induce biochemical and structural changes that allow migration. E. histolytica trophozoites exposed to 100 ng/ml of IFN-γ showed a significant increase in migration that was 4 times greater (Fig. 8Ac and Fig. 8B) than the migration of untreated control trophozoites in medium only (Fig. 8Aa and Fig. 8B; Movie S1 in the supplemental material). For these studies, IL-8 was used as a positive control (Fig. 8Ab) and cytochalasin D, an inhibitor of actin cytoskeleton, as a negative control (Fig. 8Ad). Chemotaxis of E. histolytica was also analyzed by time-lapse video. For each recording in real time, a representative E. histolytica trophozoite was selected for each condition, and their trajectories were followed throughout 4 min (Movies S1 and S2).

### IFN-γ increases E. histolytica’s cytopathic activity toward Caco-2 and HepG2 cells.

**FIG 9:**
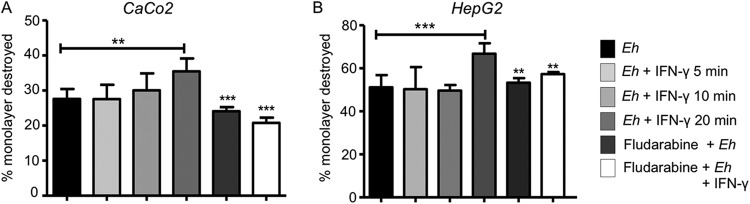
IFN-γ enhanced E. histolytica’s cytopathic effects on Caco-2 and HepG2 cells. (A, B) Caco-2 cells (A) and HepG2 cell monolayers (B) were exposed to E. histolytica trophozoites pretreated with IFN-γ at different times with or without the STAT1 inhibitor fludarabine (50 μM). Following interaction with E. histolytica, the remaining cells were stained with methylene blue and the released color measured at 655 nm. Data represent values from 4 independent experiments. **, *P* < 0.01; ***, *P* < 0.001.

The ability of E. histolytica trophozoites to destroy target cells can be separated into two phases: recognition and adhesion, which allows cell death and phagocytosis (28). To determine if IFN-γ pretreatment increased E. histolytica’s cytopathic activity, studies were carried out using human colonic Caco2 and liver HepG2 cell monolayers. After 20 min of interaction with IFN-γ, E. histolytica trophozoites had destroyed a greater percentage of the HepG2 monolayer (Fig. 9B) than of the Caco-2 cells (Fig. 9A), and the Caco-2 cell destruction was partially inhibited by the STAT1 inhibitor fludarabine. Shorter incubation times with IFN-γ and the STAT1 inhibitor had no effect on E. histolytica’s cytopathic activity.

### *In vivo* effect of stimulating E. histolytica trophozoites with IFN-γ.

**FIG 10:**
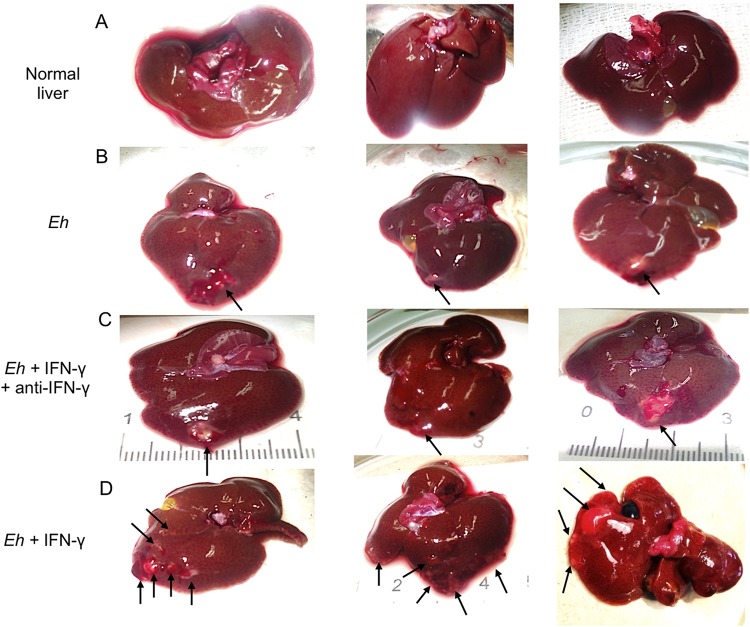
Amebic liver abscesses (ALA) 4 days postinoculation. Macroscopic development of ALA in male hamsters inoculated with E. histolytica trophozoites with or without stimulation with IFN-γ for 20 min. (A) Control uninfected livers of hamsters. (B) Single lesions (arrows) are seen in the liver lobes inoculated with E. histolytica. (C) The lesions (arrows) in the livers of hamsters treated with IFN-γ and anti-IFN-γ antibody appear similar to the ALA formed by control E. histolytica trophozoites, shown in panel B. (D) Morphology of ALA when E. histolytica trophozoites were pretreated with IFN-γ, demonstrating several granulomas distributed in the left and right lobes of the livers (arrows).

**FIG 11:**
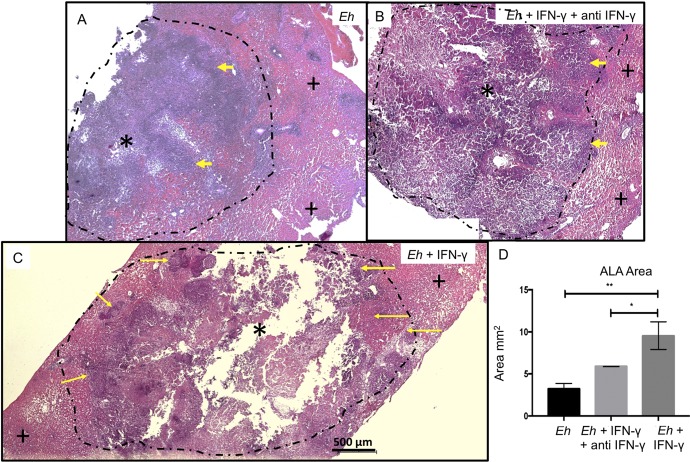
(A) Normal development of ALA. Necrotic tissue is seen in the center of the lesion (*), and the edges show areas of inflammatory infiltrate (arrowheads) that are in contact with healthy areas of the liver (**+**). (B) ALA formed by E. histolytica trophozoites in the presence of IFN-γ–anti-IFN antibody complex: the distribution of necrotic area and inflammatory infiltrates is similar to those of ALA induced by E. histolytica trophozoites. (C) ALA formed by E. histolytica trophozoites preincubated with IFN-γ (100 ng/ml). Note increased ALA development with multiple zones of new inflammatory infiltrate (yellow arrows) at the periphery of the lesions. The black dashed-line circles indicate the zones of ALA development. Hematoxylin-and-eosin staining was used. (D) Quantification of ALA development. One-way ANOVA and Tukey’s *post hoc* test were used. *, *P* < 0.05; **, *P* < 0.01.

**FIG 12:**
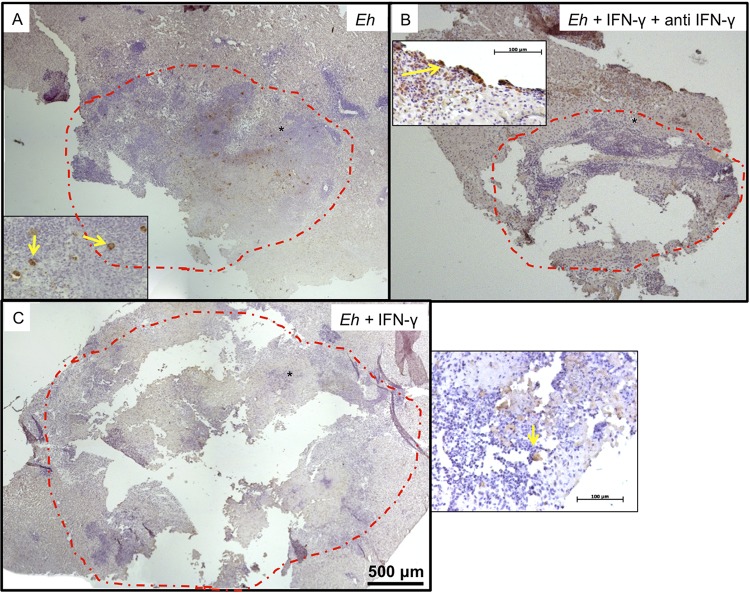
Immunohistochemical detection of E. histolytica trophozoites in hamster ALA. (A) ALA formed by control E. histolytica trophozoites. (B) ALA formed by E. histolytica trophozoites treated with IFN-γ–anti-IFN-γ complex. (C) ALA formed by E. histolytica trophozoites treated with IFN-γ. The red dashed-line circles indicate the zones of ALA development. In insets, the yellow arrows show E. histolytica trophozoites in liver tissues. The asterisk indicates the location zone of E. histolytica trophozoites.

We next tested whether treating E. histolytica with IFN-γ affected the morphological development of amebic liver abscesses (ALA) in hamsters. Hamsters were inoculated with 5 × 10^5^ E. histolytica trophozoites pretreated or not with IFN-γ for 20 min. Livers of animals inoculated with untreated trophozoites showed the characteristic ALA lesions produced after 4 days, which were small and white and localized to the site of inoculation in the left liver lobe (Fig. 10B, arrows). In marked contrast, ALA produced by trophozoites pretreated with IFN-γ, but not by trophozoites treated with IFN-γ and anti-IFN-γ antibody, produced several granulomas that were distributed throughout the left liver lobe, with a few metastatic foci in other liver lobes (Fig. 10C and D). By histopathology, the lesions produced by untreated trophozoites and trophozoites treated with IFN-γ and anti-IFN-γ antibody showed characteristic ALA granulomas that had a central necrotic region with edges of intense inflammatory infiltrates bordering healthy liver tissues (Fig. 11A and B). The lesions produced by E. histolytica trophozoites pretreated with IFN-γ (Fig. 11C) show significantly larger areas of necrosis (Fig. 11D), with several peripheral small granulomas (Fig. 11C, yellow arrows). By immunohistochemistry, trophozoites are readily seen at the periphery of the lesions (Fig. 12A and B, yellow arrows), and in lesions caused by trophozoites pretreated with IFN-γ, trophozoites were found in the lesion walls and in close proximity to healthy liver tissues (Fig. 12C, inset, yellow arrow).

## DISCUSSION

In the host-parasite interactions between E. histolytica trophozoites and human tissues, the mechanisms that trigger E. histolytica invasion are poorly understood. Our study reveals that E. histolytica has a surface protein analogous to human IFN-γ receptor 1 (IFN-γR1) and responds to the presence of IFN-γ to modify its behavior/virulence. In particular, IFN-γ activated E. histolytica trophozoites to upregulate the expression of virulence genes *EhCP-A1*, *EhCP-A2*, *EhCP-A4*, *EhCP-A5*, *APA*, *Prx*, *Hgl*, and *Cox-1*. These findings are remarkable and lend support to the idea that E. histolytica uses IFN-γ in tissues to sense inflammatory/immune cells so as to enhance invasion and/or evade immune responses. As IFN-γ is present at sites of E. histolytica invasion, in the presence of inflammatory macrophages and T cells, evading the immune response by upregulating cysteine proteases to degrade cytokines might be the most likely scenario. E. histolytica avoids the host immune response through adherence, cytotoxicity, or phagocytosis of inflammatory cells to downregulate immune effector cells and by degrading antibodies and complement with an arsenal of molecules, including amebapores, adhesins, phospholipases, serine-rich proteins, and lipopeptidophosphoglycan-like molecules (29–31). In addition, E. histolytica produces inhibiting factors that modulate immune cell function, including a monocyte locomotion inhibitory factor that prevents chemotaxis and the activation of macrophages (32). E. histolytica also produces L220, which stimulates macrophages to produce IL-10 (33), and a specific parasite-encoded cytokine, E. histolytica MIF, as a mediator of mucosal inflammation (34).

During amebic invasion in the colon, E. histolytica trophozoites are highly positive for IFN-γ located in the submucosa at sites where macrophages, γδ T cells, and NK cells are actively producing IFN-γ (35). IFN-γ activates macrophages and neutrophils that kill E. histolytica *in vitro* (35, 36), and high levels of IFN-γ have been related to resistance to intestinal amebiasis in children (37). In tissues, E. histolytica evades host immune cells by phagocytosis or apoptosis (38), by trogocytosis (39), or by inhibiting IFN-γ production with prostaglandin E_2_ (PGE_2_) from the cyclooxygenase (Cox)-like enzyme (5, 40, 41). By Western blot analysis, using an anti-human IFN-γR1 antibody, we detected the presence of an E. histolytica protein of ∼200 kDa in membrane fractions. This suggests that IFN-γ produced at the site of lesions is likely to bind the 200-kDa E. histolytica protein. By sequencing and *in silico* analysis, the 200-kDa protein showed similarity to a putative tyrosine kinase protein, with 50% homology in amino acid and physicochemical characteristics to the motif that binds human IFN-γ receptor.

The binding between IFN-γ and human immune cells is well characterized (42) for functional activity, including the release of various proinflammatory mediators critical in inflammation. Our finding of an IFN-γ receptor-like protein on the E. histolytica membrane with homology to the binding motif of human IFN-γ receptor is remarkable. Consistent with an IFN-γ receptor-like protein, exposure of E. histolytica trophozoites to IFN-γ increased their phagocytosis of erythrocytes by 15 times in a STAT1-dependent manner and upregulated several virulence genes that enhance adherence to host cells and proteases that degrade extracellular substrates. Equally important was the finding of chemotaxis of E. histolytica trophozoites toward IFN-γ, which demonstrates that E. histolytica can sense proinflammatory cytokines in lesions. Similar findings have been described for E. histolytica sensing IL-8 and TNF-α (21, 22). IFN-γ also significantly increased E. histolytica’s cytotoxicity against Caco2 and HepG2 cells. This effect may be related to increased expression of *Eh*CPs and upregulation of the Gal/GalNAc lectin to allow the parasite to disrupt innate mucosal barriers (29, 30, 43). ALA formation in hamster livers showed that E. histolytica trophozoites exposed to IFN-γ became more virulent, resulting in greater lesion sizes with multiple granulomas and metastatic foci to other liver lobes. Increased ALA formation due to preexposure of E. histolytica trophozoites to IFN-γ is a novel observation and perhaps is a strategy used by E. histolytica to bind to and deplete IFN-γ in ALA lesions. This is especially important as IFN-γ can activate newly recruited inflammatory macrophages and neutrophils to sites of infection, which is detrimental to E. histolytica. Thus, the ability of E. histolytica to bind and increase cytotoxicity toward inflammatory cells represents a strong immune evasion strategy by the parasite. In summary, our findings show that E. histolytica expresses a functional protein similar to human IFN-γR1 that functions by upregulating several key virulence factors that enhance erythrophagocytosis and cytotoxicity of epithelial cells and are critical in disease pathogenesis and immune evasion.

## MATERIALS AND METHODS

### E. histolytica culture.

E. histolytica HM-1:IMSS trophozoites were grown axenically in TYI-S-33 medium containing 10% bovine serum and supplemented with penicillin (100 U/ml), streptomycin (100 μg/ml), and 10% heat-inactivated adult bovine serum, as previously described (43). To maintain virulence, trophozoites were regularly passed through golden hamster (Mesocricetus auratus) livers as described previously (44). Trophozoites were harvested in the logarithmic phase of growth (48 to 72 h) by cooling the culture tubes on ice and centrifuging the cell suspensions at 300 × *g* for 15 min. Recovered E. histolytica trophozoites were suspended in culture medium without serum, pH 7.2, for the assays.

### Immunofluorescence detection of IFN-γ fixed on E. histolytica trophozoites.

For double immunofluorescence assays, E. histolytica trophozoites (2 × 10^5^) were cultured in coverslips in a petri dish with TYI-S-33 medium (Trypticase, yeast extract, iron serum) for 15 min at 37°C. Coverslips were washed three times with phosphate-buffered saline (PBS), medium supplemented with IFN-γ (100 ng/ml; Peprotech, Rocky Hill, NJ, USA) was added, and the trophozoites were incubated for 20, 60, or 180 min at 37°C. Coverslips were washed three times with PBS, and the trophozoites were fixed with 4% paraformaldehyde for 20 min and blocked with 10% fetal bovine serum in PBS for 1 h at 37°C. The preparations were incubated overnight at 4°C with primary antibodies anti-IFN-γ antibody (1:100) (catalog number 500-P32; Peprotech) and anti-IFN-γR1 antibody (1:100) (catalog number MA5-16583; Thermo Fisher Scientific). After three washes with PBS, secondary antibodies (1:1,000) Alexa Fluor 594-conjugated goat anti-mouse IgG(H+L) (catalog number A11005; Thermo Fisher Scientific) and Alexa Fluor 488-conjugated goat anti-rabbit IgG(H+L) (catalog number A11008; Thermo Fisher Scientific) were added and the preparations were incubated overnight at 4°C. Nuclei were stained with Hoechst stain (1 μg/ml; Thermo Fisher Scientific) in PBS for 10 min at room temperature. Coverslips were mounted with Vectashield (Vector Laboratories, Burlingame, CA, USA) and analyzed in an LMS700 microscope (Zeiss). Images were processed with ZEN 2009 Light Edition software (Zeiss). To quantify colocalization, 1-μm z-stacks of entire cells or an area around the plasma membrane were analyzed according to the Costes methodology, which includes the elimination of autofluorescence with the Coloc2 plugin of Fiji software (ImageJ). Graphing was done with GraphPad Prism 7 software (45).

### Western blot analysis for detecting IFN-γR1 on E. histolytica trophozoite membranes.

E. histolytica trophozoites were cultured in the presence or absence of 100 ng/ml of recombinant IFN-γ (Peprotech, Rocky Hill, NJ, USA) for 20 min at 37°C. After incubation, trophozoites were washed with PBS. For protein extraction, approximately 1 × 10^6^ cells were resuspended in 1 ml of lysis buffer with protease inhibitors (50 mM Tris-HCl, pH 6.8, 5 mM *N*-ethylmaleimide, 3 mM iodoacetamide, 1 mM phenylmethanesulfonyl fluoride, and 3 mM tosyl-l-lysine chloromethyl ketone). All inhibitors were purchased from Sigma-Aldrich. The cells were homogenized with −70°C/37°C incubation cycles. The lysates were centrifuged at 40,000 × *g* for 1 h at 4°C. The supernatant and pellet correspond to the cytosolic and membrane fraction, respectively. Positive-control human leukocyte total lysates were collected as well. Then, the pellets were suspended in 200 μl lysis buffer and 2.5% Triton X-100. Protein quantification was performed with the Bradford method (46). For Western blotting, 50 μg of each protein extract was separated in a 10% SDS-PAGE gel, and proteins were transferred to polyvinylidene difluoride (PVDF) membranes (Bio-Rad, Hercules, CA, USA). The membranes were blocked with Tris-buffered saline (TBS) and 5% skimmed milk for 1 h at room temperature. For immunodetection, the membranes were incubated for 24 h at 4°C with the primary antibody, a mouse anti-IFN-γR1 monoclonal antibody (1:1000; Thermo Fisher Scientific, Waltham, MA, USA). Blots were incubated with goat anti-mouse IgG conjugated with Alexa Fluor 594 (1:5,000; Millipore, Burlington, MA, USA). After the incubation, the membranes were washed with TBST (Tris-buffered saline–0.05% Tween 20) and blots were developed with Clarity Western ECL substrate (Bio-Rad, Hercules CA, USA) for chemiluminescence imaging.

### Isolation of total RNA and qPCR.

Total RNA was isolated from 1 × 10^6^ E. histolytica trophozoites incubated or not with IFN-γ for 0, 10, 20, 30, and 60 min, using the SV total RNA isolation system (Promega, Madison, WI, USA) according to the manufacturer’s protocol. Reverse transcription was performed with 50 ng of total RNA from E. histolytica trophozoites using the GoScript reverse transcription system (catalog number a5001; Promega), followed by real-time quantitative PCR (qPCR) analysis using qPCR GreenMaster with UNG, clear (Jena Bioscience, Jena. Germany) in a StepOne machine (Applied Biosystems) with the following program: 50°C for 2 min, 95°C for 3 min, and 40 cycles of 95°C for 30 s and 60°C for 30 s. Oligonucleotides were designed to target genes encoding E. histolytica cysteine proteases 1, 2, 4, and 5, amebapore, Cox-1, Gal/GalNAc lectin, and peroxiredoxin (Table S1 in the supplemental material). Relative expression levels were normalized against the expression of the respective E. histolytica genes from cells cultured without stimuli as an internal control, and differences were determined by employing the ΔΔ*C_T_* relative method. The rRNA gene was used as the housekeeping gene, using the StepOne machine (Applied biosystem).

### Erythrophagocytosis.

E. histolytica trophozoites were cultured in glass tubes under axenic conditions. Trophozoites (2 × 10^6^) were harvested by chilling the culture tubes at 4°C in a water-ice bath for 10 min and then centrifuging them at 300 × *g* for 5 min and washing with sterile Dulbecco’s PBS (DPBS) (catalog number D8537; Sigma-Aldrich). To abrogate signaling via IFN-γR1, trophozoites were pretreated with the STAT1 inhibitor fludarabine at 50 μM (catalog number Sc-204755; Santa Cruz) for 30 min before exposure to IFN-γ (100 ng/ml) for 20 min. Following incubation, trophozoites were washed twice with DPBS before the assay. Fresh human erythrocytes in DPBS solution were stained with PKH26 (catalog number MINI26; Sigma-Aldrich), counted, and used at a 1:100 (trophozoites/erythrocytes) ratio. To establish the interaction, erythrocytes were added, and the interaction was carried out for 20 min at 37°C in DPBS, after which they were washed twice with DPBS. Lysis buffer (red blood cell [RBC] lysing buffer) (catalog number R7757; Sigma-Aldrich) was added for 1 min at room temperature. Fetal bovine serum (FBS; 0.5 ml) (catalog number 10437-028; Gibco) was added for 1 min at room temperature, and the cells were washed once with DPBS. The cells were fixed with 4% paraformaldehyde for 20 min at room temperature and washed with DPBS, removing as much liquid as possible from the sample. To each tube was added a drop of FluorSave reagent (catalog number 345789; Calbiochem), and the contents were mixed carefully, placed on a clean slide, and stored in the dark at 4°C for at least 24 h before being observed under a microscope.

### Immunoprecipitation.

E. histolytica trophozoites were cultured in glass tubes under axenic conditions. Trophozoites were harvested by chilling the culture tubes at 4°C in a water-ice bath for 10 min, and then they were centrifuged at 300 × *g* for 5 min and washed with sterile DPBS (Sigma-Aldrich). Trophozoites were treated with fludarabine (50 μM) at 37°C for 30 min and then stimulated with IFN-γ (100 ng/ml) at 37°C for 20 min. Lysates were prepared from trophozoites by using a modified protocol (47). E. histolytica trophozoites were washed twice in cold PBS and suspended in radioimmunoprecipitation assay (RIPA) buffer containing protease inhibitor cocktail (Sigma), phenylmethylsulfonyl fluoride (PMSF), sodium fluoride, and sodium orthovanadate. Lysates were centrifuged at 10,000 × *g* for 10 min. Immunoprecipitation was carried out using 200 μg of protein, and the primary antibody (anti-phospho-Tyr antibody, 1.5 μg; BD transduction laboratories) was incubated with the supernatant for 8 h at 4°C, followed by 12 h at 4°C with protein A/G plus-agarose (Santa Cruz Biotechnology). Samples were run on 7.5% SDS-PAGE gels and probed with antibodies as follows: anti-STAT1 antibody (Santa Cruz) and anti-phospho-STAT1 antibody (Abcam).

### Chemotaxis.

Chemotaxis *of* E. histolytica trophozoites toward IFN-γ was monitored in Transwell migration chambers (catalog number 3464; Corning) as previously reported (48). The chemoattractant gradients were generated by placing 600 μl of culture medium without serum and containing 100 ng/ml of IFN-γ in the lower chamber of the migration units and 30,000 E. histolytica trophozoites on the filter of the upper Transwell chamber. Cells were maintained under 0.05% CO_2_ conditions at 37°C for 30 min. Cells that migrated toward IFN-γ and reached the lower chamber were recovered, stained with crystal violet, and quantified in an inverted microscope. To inhibit chemotaxis toward IFN-γ, trophozoites were treated with cytochalasin D, which depolymerizes actin/myosin filaments, as previously described (21). A coverslip chemotaxis gradient assay was used to visualize individual migrating E. histolytica trophozoites; for this, 180 μl of serum-depleted medium containing 100 ng/ml of IFN-γ was injected into a 0.75% agarose strip placed along one edge of each coverslip. On the opposite edge, trophozoites were placed along a narrow band and allowed to attach to the glass for 20 min. Chemotaxis of trophozoites toward IFN-γ (100 ng) was visualized using phase-contrast video microscopy. Video registers were made with a Carl Zeiss Axiovert 40CFL inverted microscope and the digital camera used in the immunofluorescence assays. Time-lapse videos were generated from 114 spaced frames, with 2 s between each frame acquired for 4-min real-time registers. Each video was processed with AxioVision 40V 4.6.3.0 software. To register random motility of trophozoites in the absence of IFN-γ, agarose strips not containing IFN-γ and only injected with serum-free culture medium were placed on one edge of coverslips, and migration was recorded as described above. The data analysis was performed using ImageJ 1.51n (http://imageJ.nih.gov/ij).

### Cytotoxicity assay.

Human adenocarcinoma Caco-2 cells and human liver cancer HepG2 cells from ATCC were grown to confluent monolayers in Dulbecco’s modified Eagle’s medium (DMEM) (catalog number 11995-040; Thermo Fisher) with 10% serum, penicillin, and streptomycin for 3 days (modified from the procedures in references 49 and 50). Cells grown in 24-well plates to 80 to 90% confluence were washed twice with DPBS, and 500 μl of DMEM medium was added per well. E. histolytica trophozoites (1 × 10^5^ per well) were treated or not with the STAT1 inhibitor fludarabine (50 μM) (catalog number Sc-204755; Santa Cruz) for 30 min prior to IFN-γ (100 ng/ml) stimulation for 20 min. For the interaction, trophozoites were added to each well for 1 h. After incubation, the cells were placed on ice, washed with DPBS, and fixed with 2.5% paraformaldehyde for 10 min. The monolayer was stained with 0.1% methylene blue in 0.1 M borate buffer (pH 8) for 10 min at room temperature. The plates were washed with 0.1 M borate buffer twice to remove excess stain. Finally, 1 ml of 1 N HCl was added to each well for 30 min at 37°C to extract the stain, and the absorbance read on a spectrometer at 655 nm (optical density at 655 nm [OD_655_]). The percentage of monolayer destruction was calculated as follows: [OD_655_ (control wells) − OD_655_ (experimental wells)/OD_655_ (control wells)] × 100.

### Animals.

Male golden hamsters (Mesocricetus auratus) weighing 140 to 160 g were used in this study. The animals were maintained on standard diet with free access to drinking water. All animals received humane care according to the guidelines of the Committee on Bioethics in the animal facilities of the Autonomous University of Aguascalientes, Aguascalientes, Mexico, which are based on the guidelines for animal research published by the National Institutes of Health (51).

### Experimental hepatic amebiasis.

E. histolytica trophozoites (5 × 10^5^) were incubated in the presence or absence of 100 ng/ml of recombinant IFN-γ (Peprotech, Rocky Hill, NJ, USA) for 20 min. After incubation, trophozoites were washed with PBS and inoculated into the left liver lobe of hamsters in 100 μl of culture medium as previously described (44, 52). After 4 days, the animals were anaesthetized with sodium pentobarbital (50 mg/kg of body weight intraperitoneally), the liver was excised for macroscopic evaluation, and several specimens that included ALA were taken, fixed in 4% paraformaldehyde, and processed for histological analysis.

### H&E staining.

To visualize ALA development, we performed hematoxylin and eosin (H&E) staining as described in the *Manual of Histologic Staining Methods of the Armed Forces* (53). The sample tissues were analyzed to determine the percentages of necrotic and inflammatory areas using ImageJ software.

### Immunohistochemistry.

To visualize the presence of E. histolytica trophozoites in liver tissue, sections were subjected to immunohistochemistry as described previously (53). For E. histolytica immunodetection, the samples were incubated with the primary antibody rabbit anti-E. histolytica antibody, raised in our laboratory, diluted 1:400 at 4°C. The secondary antibody, goat anti-rabbit IgG, was diluted 1:500 (Sigma-Aldrich) and incubated for 2 h at room temperature. Slides were washed with PBS-Tween 20 for 10 min, and peroxidase activity was developed with diaminobenzidine (DAB) (Pierce Biotechnology, Inc., Rockford, IL, USA) for 5 min.

### Statistical analysis.

Statistical analyses were performed using GraphPad Prism version 5.01 (GraphPad Software, San Diego, CA, USA). Student’s *t* test and one-way analysis of variance (ANOVA) with Tukey’s *post hoc* test were used. Differences between groups were assessed as significant at a *P* value of <0.05. Experimental results are represented in the figures as the mean values from three independent experiments ± standard deviations (SD).

## Supplementary Material

Supplemental file 1

Supplemental file 2

Supplemental file 3
