# Supplementary material for: Functional Characterization of an Interferon Gamma Receptor-Like Protein on Entamoeba histolytica
Source: Infect Immun. 2019 Oct 18;87(11):e00540-19. doi: 10.1128/IAI.00540-19 (PMC6803330; doi:10.1128/IAI.00540-19)
Supplement: Supplemental file 1 [file IAI.00540-19-s0001.pdf]

Supplementary **Video 1** shows motility of *Eh* and their erratic movement in short distances from a point in the absence of IFN-γ. In contrast, in Supplementary **Video 2** there is increase movement towards IFN-γ located in superior central point of video, compared to control *Eh* (Fig. 8C).
